# Supplementary material for: Electromagnetic compatibility issues in hybrid wired and wireless industrial networks
Source: PLoS One. 2020 May 1;15(5):e0232405. doi: 10.1371/journal.pone.0232405 (PMC7194443; doi:10.1371/journal.pone.0232405)
Supplement: Attachment — Submitted filename: PONE-D-19-28357_R1_rebuttal.docx [file pone.0232405.s002.docx]

Authors’ rebuttal letter

in the response to the comments and reviews on the paper
“Electromagnetic Compatibility Issues in Mixed Wired and Wireless Networked Control Systems”

submitted to PLOS ONE

**Dear Editor and Reviewers,**

Thank you for your comments; we appreciate your time and effort put in reviewing our paper.

First of all we would like to inform, that after reading the comments and considering the content we decided to change the paper title slightly. We believe the current one is more attractive and intelligible to readers. We can change it back if you conclude the first one as a better one.
The original one:
“Electromagnetic Compatibility Issues in Mixed Wired and Wireless Networked Control Systems”
The new title is:
“Electromagnetic Compatibility Issues in Hybrid Wired and Wireless Industrial Networks”

Moreover, all figures were regenerated to make them more readable. Especially, the frequency ranges were showed in the coherent values and the figures sizes are unified. Also, no raster images are currently used.

We also rearrange and updated the references section.

**In response to Additional Editor Comments:**

1) The comparison in section 3 and Table 1 is rather qualitative. The AE would like to see a more quantitative comparison, for example, how do those advantages be measured or calculated. What are their desirable ranges?

Authors’ response:
The quantitative background of this qualitative sheet is based on the analysis and conclusions made by cited Authors. The features such as dependability, spare access, and integration possibilities are not measurable. In this case they were estimated by us based on the potential ability of the given solution to make a positive impact on the given system feature. Regarding efficiency, it can be measured in various ways as throughput, time/band utilization, latency, etc. Authors cited by us in this section usually considered throughput as a measure of efficiency. In the case of [15] and [16], the increasing throughput was the goal of the cited works and the presented results confirmed the possibility of its increasing, in contrary to the other cases where the efficiency was not considered at all. EMI resistance has not been considered in the cited research, but it is the aim of our research and the results obtained confirm it can be increased.

Changes made:
We removed the schema-oriented division and depicted the research directly. Additionally, we clarified the content by a few modifications and new sentences which are all highlighted in the text.

2) The design rationales of the experiments have not been discussed in detail. How are those parameters be chosen? How closely are they reflecting the real-life scenario?

Authors’ response:
The experiment parameters and its design were based on realistic conditions reflecting the industrial applications. The frequency ranges and power have been taken from the industrial standards. In normal conditions of the industrial environment, to ensure proper operation of the whole system and its components, the disturbances should exist only in the range defined by the related standards. It is because all components comply with such standards and so, can cope with such conditions. However, in practice, due to some unusual operations, faults, event coincidences, etc., the disturbances values can go higher or interfere. The same problem exists if the disturbance does not comes from the environment but from a damage of the network infrastructure. Based on authors’ application experiences, this sometimes happens.

Changes made:
We add new paragraphs, which are all highlighted in the text. We believe they justify the design of experiments and selected parameters.

3) Some of the evaluation criteria have not been clearly elaborated. For example in Table 2, are their BER or PER?

Authors’ response:
The communication analysis has been made on application layer. It was decided because the main goal of network activity, from the system point of view, is transmission of the data entities which exist on this layer. All protocol stack layers beneath are designed for network and media operations. The real-time systems (e.g., networked control systems) and all modern heterogeneous and pervasive system models (RAMI4.0, IIRA) use networks for data transmission. A way the data is transmitted is not important regarding the system application, unless the application and time requirements, reliability and dependability are fit. Thus, in our case we analyzed the general transmission ability and we measured it by PER (ang. Packet Error Rate), where packets were sent and delivered to distributed applications. We did not analyze how the lower stack layers cope with their errors, because from the application point of view the final effect is only requested. The Profinet protocol is a well-known solution and it is well-tested. The analysis of the Profinet internal behavior and delivering BER (ang. Bit Error Rate) on the given layer could characterize only the given instrumentation and implementation of Profinet and either confirms or denies its accordance to the well-known standard. Nobody expects it works beyond the standards. We are interested in cross-standard behavior, where a hybrid network is used. Hence, our results can be used as an answer whether wired and wireless hybrid network can improve the communication, in the case when the real environment characteristics goes beyond the industrial expectations formulated in each of the constituent network standards.

Changes made:
We definitely changed the table II to make it more understandable. Additionally, we changed the description to explain the results in more detailed way.

4) What are the implications of those numbers in Table 2? Are they significant if there are error detection and correction schemes at the higher layer of the protocol stacks?

Authors’ response:
These numbers are given in the table to show clearly the disparity of the total error numbers registered in each network within the given range of frequencies. Especially, in the common range. The table does not deliver statistics analysis but only a general comparison, to ensure the reader that the difference exists and is significant. The implication is shown as a general conclusion that the hybrid network is not fault-proof, but the general EMI resistance becomes higher, if one uses it. This increase can be extremely useful in critical conditions, where EMI disturbances go beyond the limits of the given standard and application needs to keep the operational state. We use the protocol stack from the application, so the payload is either received or not. There is no access to the corrupted payload from the application. There is only access to the error detection appliance that operates on timeout. So, we either get data or not. Any additional corrections on application layer have no impact to the stack and network operation.

Changes made:
We extended the table content by showing total number of transmissions and PER value.

**Reviewers' comments:**

5. Review Comments to the Author

Reviewer #1: The manuscript presents a sound testing methodology and robust testing procedure. However, there are a number of concerns with the means by which results have been presented especially with regard to the different test cases chosen. These concerns are listed as follows:

- It is unclear for all test cases conducted how the "errors" generated are temporally distributed, that being respective error rates observed under each test condition. Results have only been presented indicating errors with respect to frequency specific interference that does not indicate the apparent impacts of said interference through a time-based measure (e.g. error rate).

Authors’ response:
Thank you for the comments.
The communication failures were detected on the real-time application level in reference to the disturbance frequency. The whole range of tested frequencies was divided into the segments according to the linear step. The error detection was performed in each frequency point during the constant time slot (time window, dwell time) of dozen seconds. It was the aim of the research. From the real-time application point of view, the most important detection is a discrete transfer status: completed/failed (error rate 0% or 100%). We assumed that even one failure violates the time constrains in a closed loop control applications and has a negative impact to the application operation. Thus, an error occurrence was the only important result and such an occurrence was tested.

During our tests we used the relatively long dwell time to increase the likelihood of catching up an error if its occurrence depends on time. Usually, the application and network loops in control systems have periods ranging from tens of microsecond up to several hundred milliseconds, so the assumed the dwell time of 12 seconds and the timeout of 1500 ms. Such values seem to be enough to perform reliable tests. Also, the error counter is added to counting up the failures occurrences in each time slot. The temporal characteristics of such error occurrences has not been collected and we have not been interested in their distribution in time. We are interested if the communication between applications is disturbed at the given frequency value at all. So, we detected errors and additionally summed them up in each segment. The number of failures characterizes communication at the given frequency during the given dwell time. Finally, the network characteristics have been obtained in this way, as a vector of EMI resistance features extracted from the consecutive frequencies and correlated measures of the failures. The temporal characteristics of error occurrence in the given frequency segment was not registered, because in our opinion it is useless from the RT application point of view. Any non-zero result means that the channel is non-operational, regarding the RT. Such characteristics could be useful, where non-RT networks and non-RT application are used. The Profinet is a deterministic protocol, where everything is scheduled and synchronized in time. It is also already well tested and standardized.

Even if we expanded the dwell time, we could collect the sets of temporal distributed failures. There are three cases arrived: 1) no errors are detected (PER 0%), 2) no transactions are completed (PER 100%), 3) some failures exist and they are distributed in time in some way (0%<PER<100%). From the real-time application point of view we expect normal operation only in case 1). The impact of cases 2) and 3) to the RT application is the same - negative. At most, in case 3) we can say that sometimes it works, sometime not, what is not allowable in the industrial domain. Thus, we cannot see the usefulness of time-based measures. The presented error rate in Table II is the summarized error rate for the whole common frequency range. It should illustrate the general difference of failures in this range to show that the wireless connection can be a backup in the case when EMI go beyond the limit defined in the standard of wired network. As we wrote in the paper, the wireless channel cannot make the hybrid network fail-proof but it can reduce the likelihood of failure.

Changes made:
We add some sentences to clarify the issue. All of them are highlighted.

- The results for each test often indicate that a threshold or "maximum" number of errors was reached for a particular dwell period at a certain point in each test. It is unclear how these errors or this quantity of errors impacts the application layer operation of the system.

Authors’ response:
In our opinion even if only one error occurs at the real-time (RT) application layer, then the RT communication can be classified as disturbed and can lead to the operational problems. If the number of errors increase during the given dwell time and at the given frequency, then the RT disturbance is much more serious. We were not interested in the impact of temporal characteristics of errors occurrence to the application layer, because regardless its shape the communication is always disturbed. One could say that the temporal characteristic of errors at the given frequency was flattening to the one point in time, which value is the measure of errors intensity. But generally, if it is greater than zero then it means the RT communication is disturbed. So, the impact is always negative. We were looking for pairs when the measure on the one network is non zero and on the second is zero, or when the measures are significantly different. Such cases occurred in some frequency ranges and proofs that use the hybrid networking has a sense from the EMI resistance point of view.

Changes made:
We add some sentences and modifications to clarify the issue. All of them are highlighted.
